# Compatible Kinetic Model for Quantitative Description of Dual-Clock Behavior of the Complex Thiourea–Iodate Reaction

**DOI:** 10.1021/acs.inorgchem.2c03594

**Published:** 2023-01-11

**Authors:** György Csekő, Qingyu Gao, Attila K. Horváth

**Affiliations:** † School of Chemical Engineering, China University of Mining and Technology, Xuzhou221116, People’s Republic of China; ‡ Department of General and Inorganic Chemistry, Faculty of Sciences, University of Pécs, Ifjúság útja 6, H-7624Pécs, Hungary

## Abstract

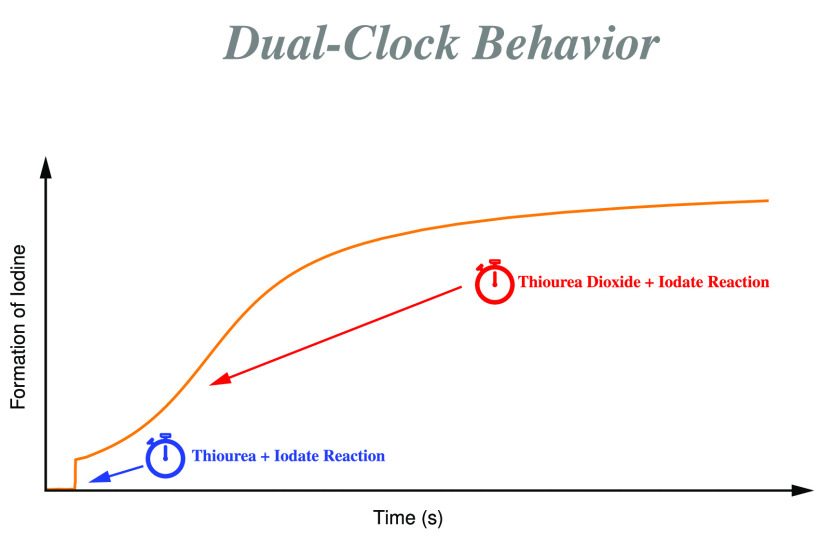

The thiourea–iodate reaction has been investigated simultaneously by ultraviolet–visible spectroscopy and high-performance liquid chromatography (HPLC). Absorbance–time traces measured at the isosbestic point of the iodine–triiodide system have revealed a special dual-clock behavior. During the first kinetic stage of the title reaction, iodine suddenly appears only after a well-defined time lag when thiourea is totally consumed due to the rapid thiourea–iodine system giving rise to a substrate-depletive clock reaction. After this delay, iodine in the system starts to build up suddenly to a certain level, where the system remains for quite a while. During this period, hydrolysis of formamidine disulfide as well as the formamidine disulfide–iodine system along with the Dushman reaction and subsequent reactions of the intermediates governs the parallel formation and disappearance of iodine, resulting in a fairly constant absorbance. The kinetic phase mentioned above is then followed by a more slowly increasing sigmoidally shaped profile that is characteristic of autocatalysis-driven clock reactions. HPLC studies have clearly shown that the thiourea dioxide–iodate system is responsible mainly for the latter characteristics. Of course, depending on the initial concentration ratio of the reactants, the absorbance–time curve may level off or reach a maximum followed by a declining phase. With an excess of thiourea, iodine may completely disappear from the solution as a result of the thiourea dioxide–iodine reaction. In the opposite case, with an excess of iodate, the final absorbance reaches a finite value, and at the same time, iodide ion will disappear completely from the solution due to the well-known Dushman (iodide–iodate) reaction. In addition, we have also shown that in the case of the formamidine disulfide–iodine reaction, unexpectedly the triiodide ion is more reactive toward formamidine disulfide than iodine. This feature can readily be interpreted by the enhancement of the rate of formation of the transition complex containing oppositely charged reactants. A 25-step kinetic model is proposed with just 10 fitted parameters to fit the 68 kinetic traces measured in the thiourea–iodate system and the second, but slower, kinetic phase of the thiourea–iodine reaction. The comprehensive kinetic model is constituted in such a way as to remain coherent in quantitatively describing all of the most important characteristics of the formamidine disulfide–iodine, thiourea dioxide–iodine, and thiourea dioxide–iodate systems.

## Abstract 2

A comprehensive kinetic model that can describe quantitatively the complex thiourea−iodate reaction featuring a dual-clock behavior is presented. A 25-step kinetic model is constituted from the previously determined kinetic models of the thiourea−iodine, thiourea dioxide−iodine, and thiourea dioxide−iodate systems supplemented with the newly proposed mechanism of the formamidine disulfide−iodine reaction and the title reaction.

## Introduction

Thiourea and its derivatives are widely used in various areas of the physical and life sciences such as microbiology, biochemistry, medicine, chemical technology, and nonlinear dynamics. One example is the thiourea-complexed cobalt(II) ion being used successfully as a cathode catalyst for proton-exchanged membrane fuel cells,^1^ and other different derivatives of thiourea (Tu) have been reported to be advantageous materials for corrosion inhibition,^2^ anion sensors,^3^ chemosensors,^4^ and colorimetric detection.^5^ Furthermore, some thiourea derivatives have recently been found to be effective antioxidants^6^ and to exhibit promising potential as antitumor drugs.^7,8^ They can also inhibit the infection of various viruses^9^ and can serve as effective substances against Gram-positive and -negative bacteria^10^ and protozoa.^11^

Oxidation of substituted Tus like methyl-thiourea, 1,3-dimethyl-thiourea, trimethyl-thiourea, tetramethyl-thiourea, *N*-acetyl-thiourea, 1-methyl-2-thiourea, phenylthiourea, and guanyl-thiourea by acidic bromate,^12−16^ chlorite,^17−20^ and iodate^21^ exhibits characteristic clock behavior, and depending on whether the substrate–clock species reactions are fast or moderate, these systems may be classified as either substrate-depletive or autocatalysis-driven clock reactions.^22^ Even though the kinetic models of these systems seem to be well-elaborated, special circumspection may sometimes reveal a questionable conclusion. For instance, in the case of the trimethyl-thiourea–chlorite reaction,^18^ the proposed model is deficient because several rate coefficients are not reported, which prevents the proper quantitative analysis of the clock behavior. Furthermore, a study of the 1,3-dimethyl-thiourea–bromate^16^ reaction suggests that the system has special but unnoticed dual-clock characteristics, though it is unclear which sulfur species is responsible for this feature.

Reactions of thiourea provide various temporal and spatial patterns; for example, the chlorite–thiourea system exhibits traveling waves^23^ and fingering patterns.^24^ When the lead(II) ion is added to the overall chlorite–thiourea system, the chemical reaction along with the hydrodynamic processes forms a complex network, resulting in the morphogenesis of an unusual precipitation pattern.^25^ The thiourea–iodate reaction perturbed by sulfite leads to spatial bistability and stationary patterns^26−28^ as well as oligooscillation^29,30^ in batch and complex oscillations^31,32^ in a continuously stirred flow reactor.

Quantitative explanation of the temporal and spatial behavior of these systems often requires a complex mechanistic background where one may easily overlook the effect of cross-coupled reactions like in the case of the autocatalytic thiourea–bromine reaction,^33^ where as a result of the fast direct thiourea–bromine reaction shown later^34^ the formamidine disulfide–bromine system is actually responsible for the observed autocatalytic behavior.

This paper attempts to report the overall kinetic model of the complex thiourea–iodate reaction that can characterize the kinetic features of the title reaction quantitatively. Although we may cite two previous independent works,^29,30^ the kinetic model of both reports contradicts our recent findings. For example, the model used for the explanation of oligooscillatory behavior uses a very simple thiourea dioxide–iodine reaction that is complex and inhibited by the product iodide ion.^35^ In addition, the proposed kinetic model contains the direct thiourea trioxide–iodine reaction that actually does not occur at all.^36^ All of the facts mentioned above motivate a detailed investigation of the title reaction to establish a comprehensive kinetic model to be used for quantitative description of the fascinating thiourea–iodate system.

## Experimental Section

### Materials

All of the reagents were of the highest purity available commercially and were used without further purification. Stock solutions were made by dissolving the necessary amount of the target compounds. Water was ion-exchanged twice and then distilled twice to remove ionic exchange resin residues and dissolved gases. All of the solutions were deoxygenated by being bubbled with oxygen free argon for at least 10 min. All of the experiments were performed at a constant ionic strength of 1.0 M by using sodium perchlorate as a background electrolyte, except when otherwise stated. In the case of the thiourea–iodate reaction, the initial thiourea, iodate, and iodide concentrations were varied in the ranges of 0.4–5.0, 0.57–4.0, and 0–0.1 mM, respectively. Upon investigation of the thiourea–iodine reaction, the initial concentrations of thiourea, iodine, and iodide varied in the ranges of 0.06–0.7, 0.4–1.3, and 0–40.0 mM, respectively. The pH in both cases was adjusted with phosphoric acid/dihydrogen phosphate buffer within the range of 2.0–3.14 using the p*K*_a1_ of phosphoric acid of 1.8,^37^ keeping the concentration of H_2_PO_4_^–^ constant at 0.25 M throughout the experiments.

### Instrumentation

The kinetic runs were carried out with a Zeiss S600 diode array spectrophotometer equipped with a thermostated cell holder, setting the temperature at 25.0 ± 0.1 °C. The thiourea–iodate system was also monitored by high-performance liquid chromatography (HPLC) to identify the key intermediates over the whole course of the reaction. The HPLC separation experiments were performed on a Thermo UltiMate 3000 instrument equipped with a DAD-3000 multiple-wavelength detector, a Phenomenex Gemini C18 separation column (5.0 μm, 46 μm × 250 mm), and a model LPG-3400SD pump with four pistons. The eluent was a mixture of a 1 mM tetrabutylammonium hydroxide (TBAOH) aqueous solution (pH ≈6.7 adjusted by dropwise addition of a phosphoric acid solution) and methanol (95 vol %) at a flow rate of 0.4 cm^3^ min^–1^.

### Treatment of Data

The absorbance–time profiles were evaluated simultaneously with ZiTa/Chemmech.^38^ An orthogonal fitting procedure was applied, meaning that all of the kinetic curves were transformed into a 0 ≤ *x*, *y* ≤ 1 box and the sum of the perpendicular deviation between the measured and calculated absorbances was minimized by kinetic parameter optimization. Our criterion was that the average deviation not exceed 2.0%, which is the experimentally achievable limit of error under our experimental conditions.

## Results and Discussion

### Combined Ultraviolet–Visible (UV–vis) and HPLC Studies

**Figure 1:**
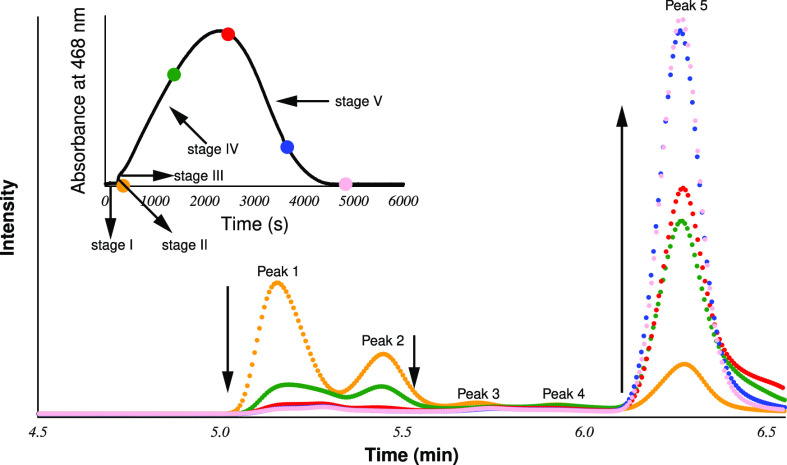
Typical absorbance–time curves (top left corner) and HPLC chromatograms measured in an excess of thiourea. Initial conditions were as follows: [Tu]_0_ = 3.38 mM, [IO_3_^–^]_0_ = 2.13 mM, pH 2.63, and [I^–^]_0_ = 0 mM. Note that here no extra background salt was applied; thus, the ionic strength was controlled mainly by the buffer component [H_2_PO_4_^–^]_0_ = 0.25 M. The colored dots denote the time points at which samples were taken from the cuvette and injected into the HPLC instrument. The color code of the chromatogram indicates the time point of taking the samples.

**Figure 2:**
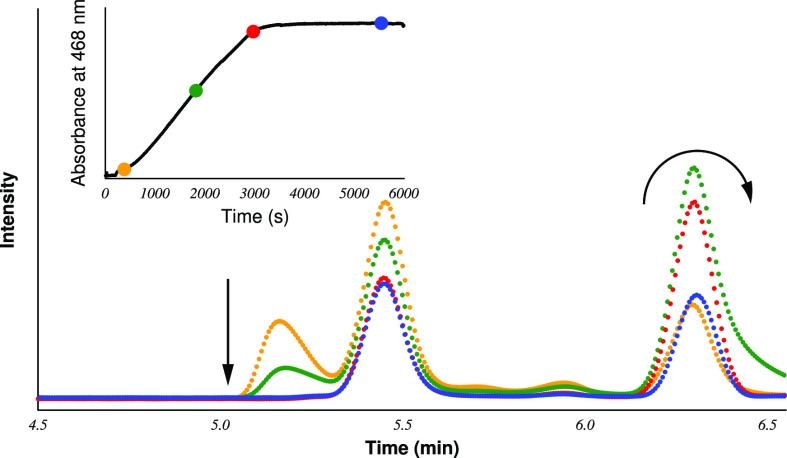
Typical absorbance–time curves (top left corner) and HPLC chromatograms measured in an excess of iodate. Initial conditions were as follows: [Tu]_0_ = 1.0 mM, [IO_3_^–^]_0_ = 2.8 mM, pH 2.43, and [I^–^]_0_ = 0 mM. Note that here no extra background salt was applied; thus, the ionic strength was controlled mainly by the buffer component [H_2_PO_4_^–^]_0_ = 0.25 M. The colored dots denote the time points at which samples were taken from the cuvette and injected into the HPLC instrument. The color code of the chromatogram indicates the time point of taking the samples.

The top left corner of Figure 1 depicts a typical but complex absorbance–time trace measured at the isosbestic point of the iodine–triiodide system (468 nm, where ε_I_2__ = ε_I_3_^–^_ = 750 M^–1^ cm^–1^)^39^ in a stoichiometric excess of thiourea. The profile may easily be separated into several stages as shown below. As it is visualized, the reaction starts with a fairly long induction time where iodine does not appear at all (stage I). When this period is over, iodine forms abruptly (stage II), and soon its formation slows, resulting in an inflection point or a longer apparently stationary state on the kinetic curve (stage III). The system is slowly transferred into the next phase when the total iodine concentration starts to increase further (stage IV), reaches a maximum, and then decreases (stage V). To clarify the key reactions governing these stages, samples were taken from the cuvette at the time points indicated by colored circles and a systematic HPLC analysis was performed in each case. As a result, the time evolution of the HPLC chromatogram is also plotted in Figure 1. As one can clearly see, three major and two minor peaks appeared with different tendencies. The height of the major peaks enumerated by 1 and 2 monotonically decreases with time, while that of the major peak designated by 5 monotonically increases. The trends of the minor peaks (3 and 4) are difficult to see, though it looks to be clear that they belong to key intermediates of the reaction. Separate HPLC experiments showed that the peaks of formamidine disulfide (FDS), iodate, thiourea dioxide, thiourea monoxide, and thiourea [along with the iodide ion (see below)] appear at 5.14, 5.45, 5.70, 5.94, and 6.29 min, respectively. (see Figure S1). From this information, it seems clear that at stage I the major sulfur-containing intermediate is formamidine disulfide forming via the rapid thiourea–iodine reaction. It may be understood that at the very beginning of this reaction, iodate is reduced to the iodide ion by thiourea via successive oxygen transfer processes. Once the iodide ion appears, it ignites the Dushman reaction to produce iodine, which is instantaneously removed by thiourea. Therefore, as long as thiourea is present, iodine cannot appear (stage I), which makes the title reaction a classic example of the substrate-depletive clock reaction.^22^ Once the substrate thiourea is totally consumed, iodine appears rapidly due to the Dushman reaction (stage II), removing a majority of the iodide ion from the solution. As a result, the iodide-autoinhibited TDO–iodine reaction and the TMO–iodine and FDS–iodine reactions start to work, which prevent the further buildup of iodine; hence, we arrive at stage III, where the absorbance remains fairly constant for a while (see Figure S5) due to the fine balance of the iodine consumption and formation reactions. When FDS and TMO are completely consumed, this balance is gradually lost. As a result, the TDO–iodate autocatalysis-driven clock reaction takes control of increasing the amount of iodine in stage IV.^40^ Once iodate is removed completely, thiourea dioxide is still present in the solution and ready to remove iodine in the fairly long thiourea dioxide–iodine reaction that was found to be autoinhibited by the iodide ion, resulting in stage V.^35^ The qualitative picture we present here is in complete harmony with the time evolution of the corresponding peaks on the HPLC chromatogram except for one important fact. If the peak at 6.29 min corresponds uniquely to thiourea, its height should not increase as the reaction proceeds. This observation suggests that there must be another species present in the system having the same retention time as thiourea. To resolve the clue, we have also analyzed the system simultaneously by UV–vis and HPLC in an excess of iodate. The result is shown in Figure 2. These measurements have revealed that the height of the peak referenced at 6.29 min goes through a maximum, supporting further that in addition to thiourea another species is responsible for its time evolution. A conceivable explanation of this behavior is that the iodide ion has the same retention time under the applied experimental condition; thus, this peak is the superposition of these species. [This suggestion was positively checked by separate experiments when just the iodide ion-containing sample was injected into the HPLC instrument under the same experimental condition (see Figure S1).] The characteristics of the maximum may easily be reconciled by the fact that even if thiourea is removed at the beginning stage of the reaction the concentration of the iodide ion increases once sulfur species reacting with elemental iodine exist in the solution. If, however, thiourea, formamidine disulfide, thiourea monoxide, and thiourea dioxide are all removed from the system, the Dushman reaction^41^ (iodide–iodate system) finally decreases the concentration of the iodide ion, resulting in a decrease in the height of the peak at 6.29 min. If the excess of iodate is large enough, then the iodide ion is completely depleted from the solution that would also correspond to the disappearance of this peak. One may clearly see that the results of the combined UV–vis and HPLC experiments provided here provide further insights (compared to those of the previously published reports^29,30^) into the unusually complex kinetic behavior of the title system, shedding light on the dual-clock behavior under batch conditions as well as the role of the long-lived intermediate during the course of the reaction.

### Limiting Stoichiometries

As Figure 2 suggests, the final stoichiometry in a large excess of iodate can be expressed by the following equation:
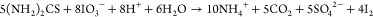
1Evidently, in this case iodine should be the lone iodine-containing product (besides the excess of iodate), and all of the sulfur-containing compounds are oxidized to sulfate. However, in the excess of thiourea, the stoichiometry cannot be expressed by a single equation because besides sulfate, thiourea dioxide may also appear as a sulfur-containing final product. Therefore, the corresponding stoichiometry under a given initial condition may be obtained by the linear combination of eqs 2 and 3:
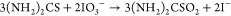
2and
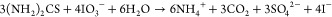
3For the sake of completeness, it should be noted that it may be impossible to find an experimental condition under which eq 2 or 3 may purely characterize the stoichiometry of the title reaction.

### Proposed Kinetic Model

**Figure 3:**
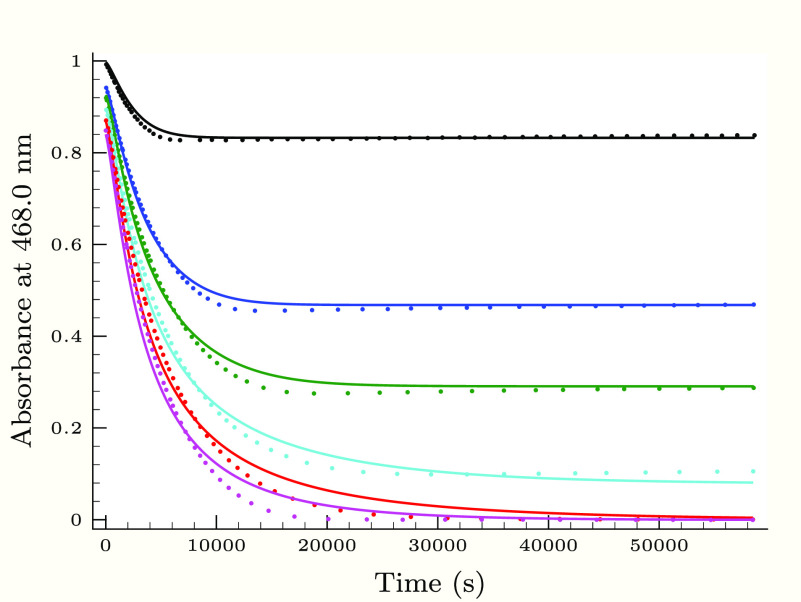
Experimental (dots) and calculated (solid lines) absorbance–time profiles in the second (slower) phase of the thiourea–iodine reaction, when the initial thiourea concentration is varied applying 0.25 M H_2_PO_4_^–^ buffer component. Initial conditions were as follows: *T*_I_2__^0^ = 1.36 mM, [I^–^]_0_ = 12.6 mM, pH 2.83, and [Tu]_0_/mM = 0.064 (black), 0.184 (blue), 0.247 (green), 0.323 (cyan), 0.399 (red), and 0.471 (magenta).

**Figure 4:**
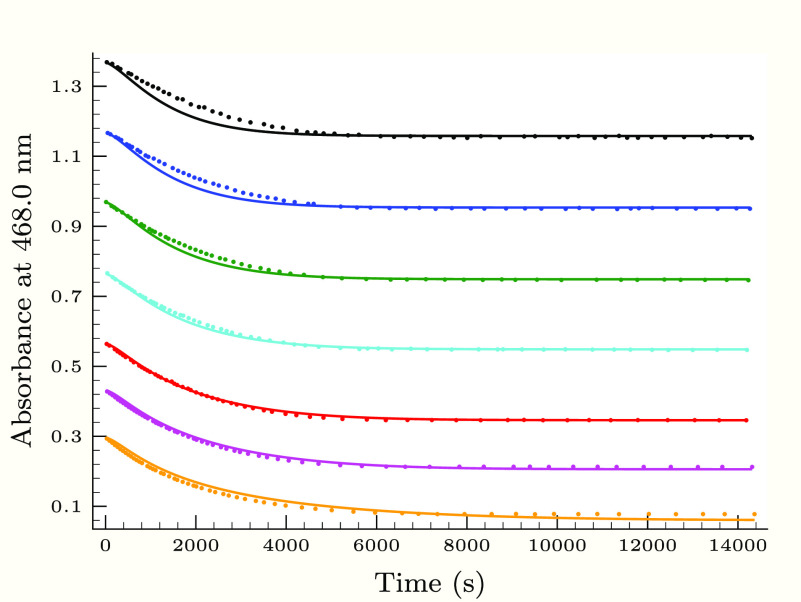
Experimental (dots) and calculated (solid lines) absorbance–time profiles in the second (slower) phase of the thiourea–iodine reaction, when the initial iodine concentration is varied applying 0.25 M H_2_PO_4_^–^ buffer component. Initial conditions were as follows: [Tu]_0_ = 0.084 mM, [I^–^]_0_ = 17.6 mM, pH 3.14, and *T*_I_2__^0^/mM = 1.87 (black), 1.60 (blue), 1.34 (green), 1.06 (cyan), 0.8 (red), 0.622 (magenta), and 0.444 (orange).

**Figure 5:**
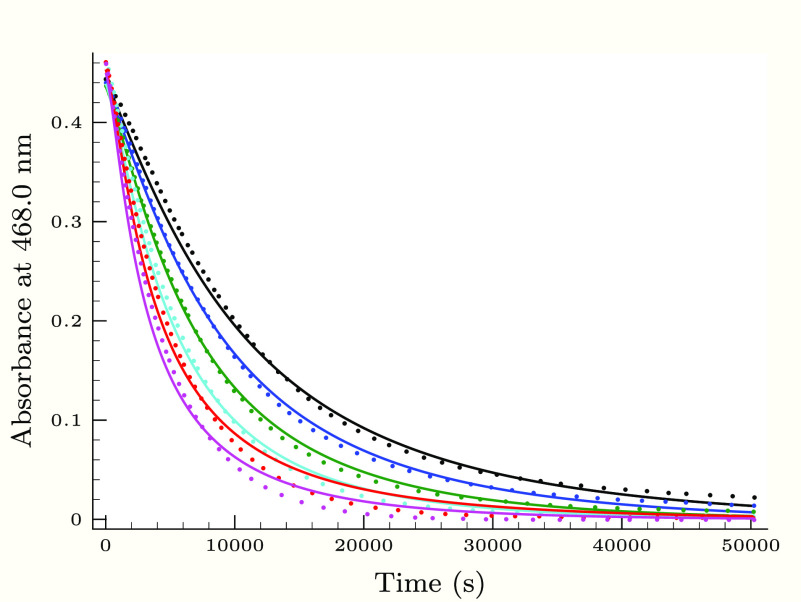
Experimental (dots) and calculated (solid lines) absorbance–time profiles in the second (slower) phase of the thiourea–iodine reaction, when the pH is varied applying 0.25 M H_2_PO_4_^–^ buffer component. Initial conditions were as follows: [Tu]_0_ = 0.24 mM, [I^–^]_0_ = 11.3 mM, *T*_I_2__^0^ = 0.72 mM, and pH 2.26 (black), 2.43 (blue), 2.63 (green), 2.83 (cyan), 3.03 (red), and 3.14 (magenta).

**Figure 6:**
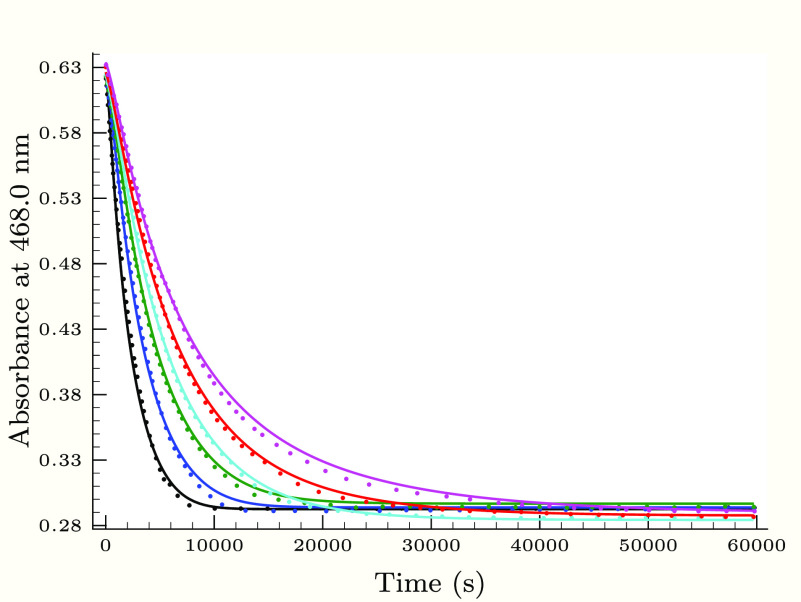
Experimental (dots) and calculated (solid lines) absorbance–time profiles in the second (slower) phase of the thiourea–iodine reaction, when the concentration of the initially added iodide ion is varied applying 0.25 M H_2_PO_4_^–^ buffer component. Initial conditions were as follows: [Tu]_0_ = 0.132 mM, pH 3.14, *T*_I_2__^0^ = 0.9 mM, and [I^–^]_0_/mM = 8.4 (black), 14.6 (blue), 20.9 (green), 27.1 (cyan), 33.4 (red), and 41.2 (magenta).

**Figure 7:**
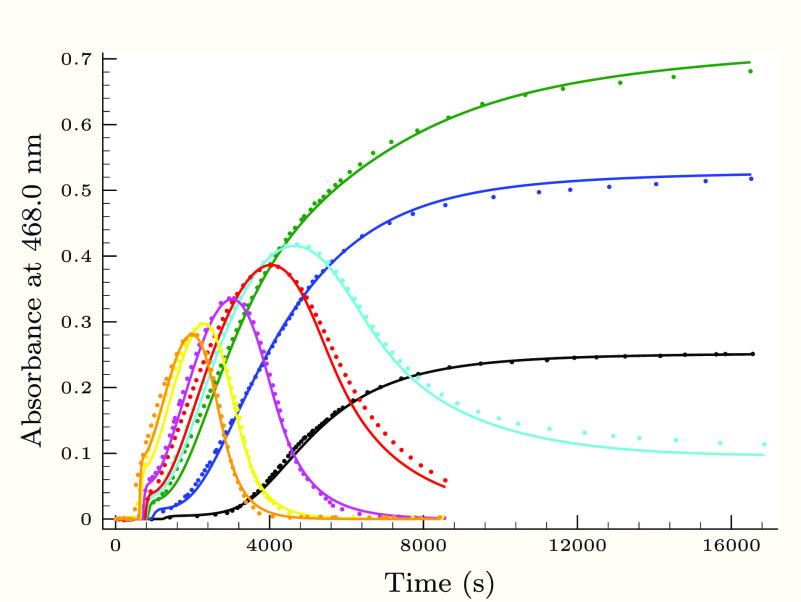
Experimental (dots) and calculated (solid lines) absorbance–time profiles in the case of the thiourea–iodate reaction, when the initial thiourea concentration was varied in the absence of initially added iodide ion applying 0.25 M H_2_PO_4_^–^ buffer. Initial conditions were as follows: [IO_3_^–^]_0_ = 2.13 mM, pH 2.63, and [Tu]_0_/mM = 0.43 (black), 0.89 (blue), 1.34 (green), 1.56 (cyan), 1.69 (red), 2.15 (magenta), 2.87 (yellow), and 3.38 (orange).

**Figure 8:**
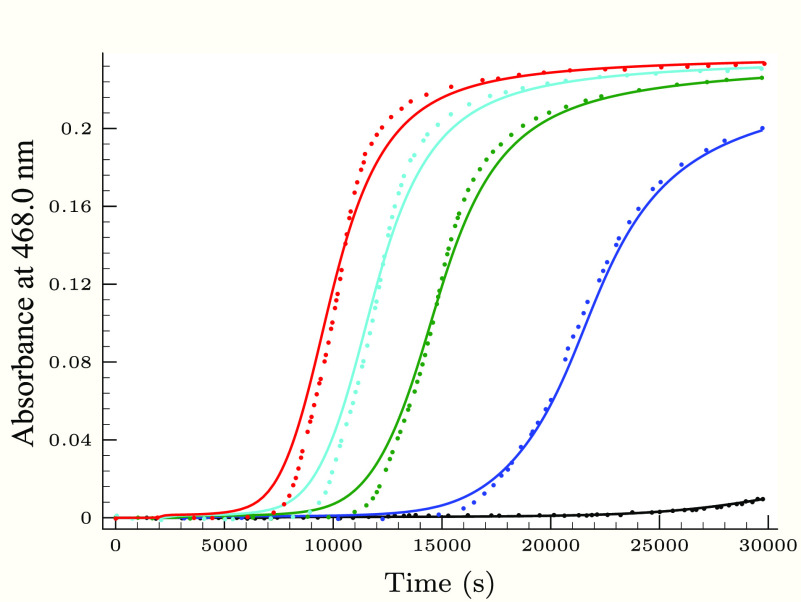
Experimental (dots) and calculated (solid lines) absorbance–time profiles in the case of the thiourea–iodate reaction, when the initial iodate concentration was varied in the absence of initially added iodide ion applying 0.25 M H_2_PO_4_^–^ buffer component. Initial conditions were as follows: [Tu]_0_ = 0.403 mM, pH 2.83, and [IO_3_^–^]_0_/mM = 0.55 (black), 0.88 (blue), 1.26 (green), 1.57 (cyan), and 1.89 (red).

**Figure 9:**
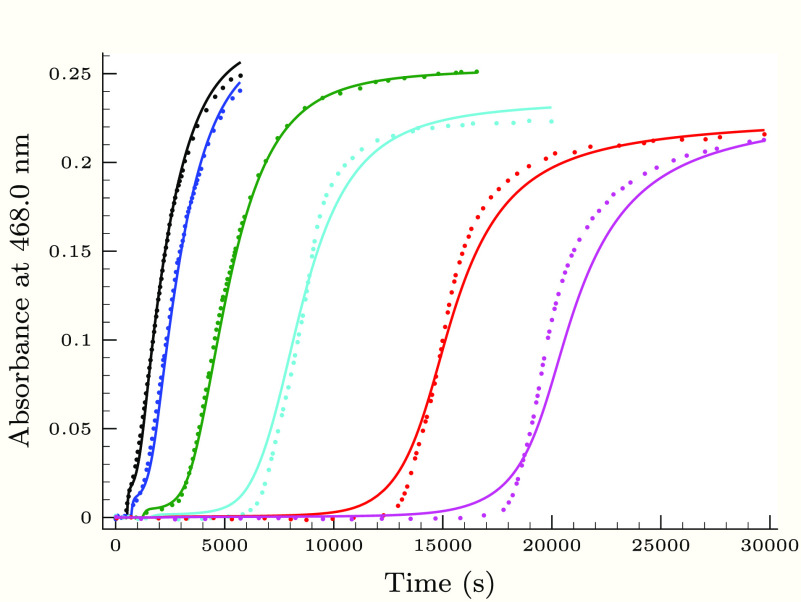
Experimental (dots) and calculated (solid lines) absorbance–time profiles in the case of the thiourea–iodate reaction, when the pH was varied in the absence of initially added iodide ion applying 0.25 M H_2_PO_4_^–^ buffer component. Initial conditions were as follows: [Tu]_0_ = 0.43 mM, [IO_3_^–^]_0_ = 2.5 mM, and pH 2.26 (black), 2.43 (blue), 2.63 (green), 2.83 (cyan), 3.03 (red), and 3.14 (magenta).

**Figure 10:**
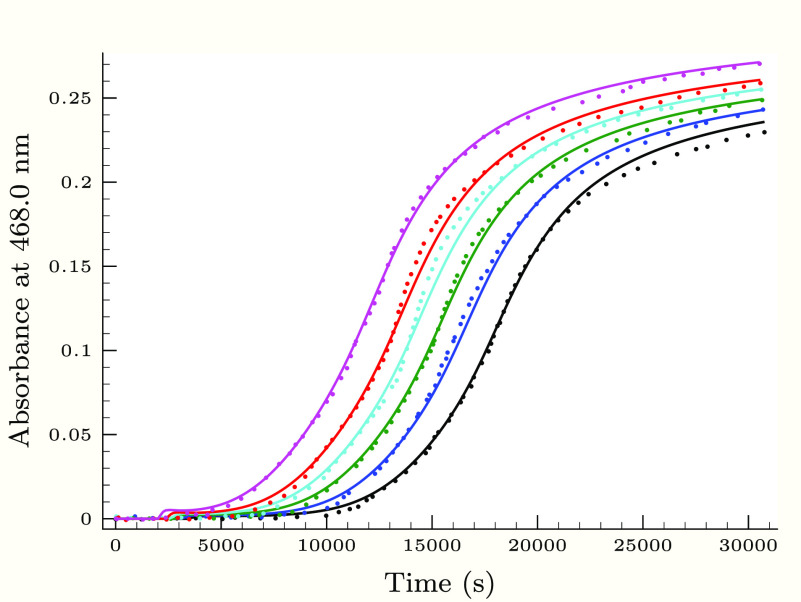
Experimental (dots) and calculated (solid lines) absorbance–time profiles in the case of the thiourea–iodate reaction, when the concentration of initially added iodide ion was varied applying 0.25 M H_2_PO_4_^–^ buffer component. Initial conditions were as follows: [Tu]_0_ = 0.45 mM, [IO_3_^–^]_0_ = 0.85 mM, pH 2.83, and [I^–^]_0_/mM = 0.02 (black), 0.03 (blue), 0.04 (green), 0.05 (cyan), 0.06 (red), and 0.08 (magenta).

Identification of the different kinetic stages of the complex thiourea–iodate reaction makes it possible to elucidate the proposed model in a stepwise fashion. The inclusion of some subsystems required either independent but short (Tu–iodine system) or extended studies (e.g., FDS–iodine system) to determine correctly the rate coefficients involved in the overall system, or simply they were inserted without any change (TDO–iodine reaction) or by slight but reasonable modification in the steps and their corresponding rate coefficients (TDO–iodate system). The comprehensive kinetic model is shown in Table 1. The average deviation between the measured and calculated data in the case of both the second kinetic phase of the thiourea–iodine and thiourea–iodate systems was found to be 1.51%, indicating sound overall agreement as Figures 3–10 suggest.

**Table 1:** Fitted and Fixed Rate Coefficients of the Proposed Kinetic Model^a^

system	no.	reaction	rate equation	parameter value	refs
preequilibria	R1	H_3_PO_4_ ⇌ H^+^ + H_2_PO_4_^–^	*k*_R1_[H_3_PO_4_]	1.58 × 10^8^ s^–1^	n.a.
			*k*_–R1_[H^+^][H_2_PO_4_^–^]	10^10^ M^–1^ s^–1^	n.a.
	R2	HIO_3_ ⇌ H^+^ + IO_3_^–^	*k*_R2_[HIO_3_]	10^10^ s^–1^	(42), (43)
			*k*_–R2_[H^+^][IO_3_^–^]	3.125 × 10^10^ M^–1^ s^–1^	
	R3	I_2_ + I^–^ ⇌ I_3_^–^	*k*_R3_[I_2_][I^–^]	5.6 × 10^9^ M^–1^ s^–1^	(44), (45)
			*k*_–R3_[I_3_^–^]	8.5 × 10^6^ s^–1^	
TDO–I_2_ reaction	R4	I_2_ + H_2_O ⇌ H^+^ + I^–^ + HOI	*k*_R4_[I_2_]	0.0552 s^–1^	(46−48)
			*k*_–R4_[HOI][I^–^][H^+^]	1.023 × 10^11^ M^–2^ s^–1^	
			*k*_R4_^′^[I_2_][H^+^]^−1^	1.98 × 10^–3^ M s^–1^	
			*k*_–R4_^′^[HOI][I^–^]	3.67 × 10^9^ M^–1^ s^–1^	
	R5	TDO + I_2_ ⇌ TDOI + I^–^ + H^+^	*k*_R5_[TDO][I_2_]	10^3^ M^–1^ s^–1^	(35)
			*k*_–R5_[TDOI][H^+^][I^–^]	10^9^ M^–2^ s^–1^	
	R6	TDOI + 3H_2_O → I^–^ + HSO_3_^–^ + CO_2_ + 2NH_4_^+^	*k*_R6_[TDOI]	114 s^–1^	(35)
	R6′		*k*_R6_^′^[H^+^]^−1^[TDOI]	0.0495 M s^–1^	
	R7	TDO → TDO_aged_	*k*_R7_[TDO]	1.87 × 10^–6^ s^–1^	(35)
	R8	I_2_ + TDO_aged_ → 2I^–^ + TDO^2+^	*k*_R8_[TDO_aged_][I_2_]	10^4^ M^–1^ s^–1^	(35)
	R9	TDO + TDO^2+^ + 4H_2_O → SO_4_^2–^ + S + 2CO_2_ + 4NH_4_^+^	*k*_R9_[TDO^2+^][TDO]	10^5^ M^–1^ s^–1^	(35)
	R10	HSO_3_^–^ + I_2_ + H_2_O → SO_4_^2–^ + 2I^–^ + 3H^+^	*k*_R10_[HSO_3_^–^][I_2_]	3.1 × 10^9^ M^–1^ s^–1^	(49)
TDO–IO_3_^–^ reaction	R11	TDO + IO_3_^–^ + 2H^+^ + 2H_2_O → HSO_3_^–^ + HIO_2_ + CO_2_ + 2NH_4_^+^	*k*_R11_[TDO][IO_3_^–^][H^+^]^2^	29.7 M^–3^ s^–1^	(39)
	R12	TDO + HIO_2_ + H^+^ + 2H_2_O → HSO_3_^–^ + HOI + CO_2_ + 2NH_4_^+^	*k*_R12_[TDO][HIO_2_]	10^6^ M^–1^ s^–1^	(39)
	R13	TDO + HOI + H^+^ + 2H_2_O → HSO_3_^–^ + I^–^ + CO_2_ + 2NH_4_^+^	*k*_R13_[TDO][HOI]	10^4^ M^–1^ s^–1^	(39)
	R14	HIO_3_ + I^–^ + H^+^ ⇌ I_2_O_2_ + H_2_O	*k*_R14_[HIO_3_][I^–^][H^+^]	10^7^ M^–2^ s^–1^	(39)
			*k*_–R14_[I_2_O_2_]	10^8^ s^–1^	(39)
	R15	H^+^ + I^–^ + I_2_O_2_ → I_2_ + HIO_2_	*k*_R15_[I^–^][I_2_O_2_]	(2.19 ± 0.09) × 10^9^ M^–1^ s^–1^	this work
	R16	H^+^ + I^–^ + HIO_2_ → 2HOI	*k*_R16_[H^+^][I^–^][HIO_2_]	10^9^ M^–2^ s^–1^	(39)
	R17	TDO_aged_ + IO_3_^–^ + 2H^+^ + 2H_2_O → HSO_3_^–^ + HIO_2_ + CO_2_ + 2NH_4_^+^	*k*_R17_[TDO_aged_][IO_3_^–^][H^+^]^2^	7608 M^–3^ s^–1^	(39)
Tu–I_2_ reaction	R18	2Tu + I_2_ → FDSH_2_^2+^ + 2I^–^	*k*_R18_[Tu][I_2_]	10^5^ M^–1^ s^–1^	fixed
hydrolysis of FDS	R19	FDSH_2_^2+^ + H_2_O ⇌ Tu + TMO + 2H^+^	*k*_R19_[FDSH_2_^2+^]	(1.07 ± 0.04) × 10^–4^ s^–1^	this work
			*k*_–R19_[Tu][TMO]	(9.32 ± 0.71) × 10^6^ M^–1^ s^–1^	this work^b^
FDS–I_2_ reaction	R20	I_2_ + TMO + H_2_O → 2I^–^ + 2H^+^ + TDO	*k*_R20_[I_2_][TMO]	50.7 ± 3.7 M^–1^ s^–1^	this work
	R21	I_3_^–^ + FDSH_2_^2+^ + 2H_2_O → 4H^+^ + 3I^–^ + 2TMO	*k*_R21_[I_3_^–^][FDSH_2_^2+^]	(2.92 ± 0.19) × 10^–4^ M^–1^ s^–1^	this work
	R22	Tu + HOI → H^+^ + I^–^ + TMO	*k*_R22_[Tu][HOI][H^+^]^−1^	(1.41 ± 0.12) × 10^9^ s^–1^	this work
	R23	TMO + H_2_O + 2H^+^ → CO_2_ + 2NH_4_^+^ + S	*k*_R23_[TMO]	(7.63 ± 0.69) × 10^–5^ s^–1^	this work
Tu–IO_3_^–^ reaction	R24	Tu + IO_3_^–^ + H^+^ → TMO + HIO_2_	k_R24_[Tu][IO_3_^–^]	0.0285 ± 0.0065 M^–1^ s^–1^	this work
			*k*_R24_^′^[Tu][IO_3_^–^][H^+^]	31.27 ± 0.05 M^–2^ s^–1^	this work
	R25	TMO + HIO_2_ → TDO + HOI	*k*_R25_[TMO][HIO_2_][H^+^]	(4.54 ± 0.38) × 10^10^ M^–2^ s^–1^	this work

a If the standard deviation appears with the rate coefficient, it means that the given parameter is fitted during the course of the evaluation process; otherwise, the given value is fixed.

b The *k*_–R19_ rate coefficient is conditional, valid for the pH range applied.

### Rapid Preequilibria

The first three rows of Table 1 contain all of the necessary but rapid preequilibria to be included in the proposed model for an adequate quantitative description of the absorbance–time traces measured in the thiourea–iodine and thiourea–iodate systems. For the sake of completeness, it should also be mentioned that in the cases of their subsystems such as the TDO–iodate and TDO–iodine ones, these equilibria are also indisputable parts for adequate modeling. These are the auxiliary protonation and deprotonation equilibria of the buffer components and that of the iodate as well as the formation of the triiodide ion. The equilibrium constants of these processes are well-known from the literature;^37^ thus, the rate coefficients of the forward and backward processes were fixed in such a way that, first, their ratio has to give the corresponding equilibrium constant and, second, all of these equilibria are established rapidly. The corresponding rate coefficients are also included in Table 1 to take the small pH change into consideration correctly during the course of the reaction. This is a crucial point, especially when the given model (like in our case) contains reactions whose rate depends strongly on pH.

### Thiourea Dioxide–Iodine Reaction

Our combined HPLC and UV–vis studies have clearly revealed that thiourea dioxide (TDO) is a key long-lived intermediate of the title reaction even when iodine is present in the system; thus, evidently the kinetic model of the thiourea dioxide–iodine reaction has to be included in the proposed mechanism. This kinetic model was already determined by a recent work^35^ under quite similar experimental circumstances; therefore, we directly included all of the steps with their corresponding rate coefficients in our model. They can also be seen in Table 1. Such a treatment allows the kinetic model established here to remain coherent; thus, it is still capable of describing quantitatively the kinetic curves measured previously in the thiourea dioxide–iodine reaction. For the sake of clarity, it should also be emphasized that part of this kinetic model may be eliminated without any significant change in the average deviation between the experimental and calculated kinetic curves of the fitting process of the title reaction. To be more precise, the sequence of the pathway belonging to the reaction of aged thiourea dioxide cannot play any significant role in the title reaction just because TDO is inherently and freshly produced during the course of the reaction. To support this statement, first, we performed experiments with aged stock solutions in the thiourea–iodate system. Figure S3 unambiguously demonstrates that indeed there is no substantial aging effect observed. In addition, we repeated the fitting procedure in which steps R7–R9 along with step R17 (see below) were excluded from the kinetic model. The average deviation was found to be 1.57%, providing further support for the idea that this route plays only a minor role in correct description of the title system. It should also be noted that under our experimental conditions sulfur precipitation (or even slight turbidity) was not observed at all. Because aging of TDO is the only route for the appearance of sulfur precipitation, the lack of this phenomenon also supports our calculations that indeed this route may easily be eliminated from the model.

### Thiourea Dioxide–Iodate Reaction

The necessity of including the kinetic model of the thiourea dioxide–iodate reaction is also a self-speaking consequence of the HPLC measurements. The experimental conditions applied here were quite close to those reported in one of our previous works,^39^ though here a lower acidity (approximately an order of magnitude lower [H^+^]) was used to monitor conveniently the title reaction. Therefore, we thought that the kinetic model of the thiourea dioxide–iodate reaction could be used without any modifications, but our calculations revealed that this assumption is just valid with almost all of the rate coefficients determined previously except for *k*_R15_. If we fixed *k*_R15_ to 4.67 × 10^8^ + 6.29 × 10^10^[H^+^] M^–1^ s^–1^ as reported elsewhere,^39^ then the average deviation between the measured and calculated data would have never decreased below 5.0%. After fitting this rate coefficient as well, at a *k*_R15_ value of (2.19 ± 0.09) × 10^9^ M^–1^ s^–1^, we obtained an average deviation of 1.51%; therefore, we decided to use this value to visualize the quality of the fit, though certainly a word is also in an order here to support this change. First, even though in the case of the iodate–arsenous acid system^50^ the following reaction was found to play an important role
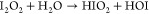
4providing an additional sequence of reactions leading to the formation of products, in the TDO–iodate reaction its significance was explicitly ruled out.^39^ Our calculation, here, confirmed our previous observation that indeed inclusion of eq 4 cannot improve the quality of the fit. Second, the *k*_R15_ rate coefficient was determined to be 1.02 × 10^9^ M^–1^ s^–1^ in the case of the iodate–arsenous acid system,^50^ and this value is approximately half of the value reported here. Considering that the iodate–iodide reaction is, indeed, the subject of specific and general acid catalysis,^51−54^ this difference can easily be reconciled by the application of phosphate buffer in the title reaction. At the same time, it indirectly supports our findings here that R15 becomes independent of pH at lower pHs.

### Thiourea–Iodine Reaction

There is a consensus in the literature about the stoichiometry of the reaction in that it can be expressed in terms of the following chemical equation^29^
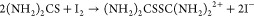
5though formamidine disulfide may slowly be transformed further into aminothiazole or aminothiadiazole in the absence of oxidants.^55^ Because formamidine disulfide was found to be an intermediate in the title system and can be oxidized further by iodate, iodine, and other reactive iodine-containing species, this slow transformation reaction may readily be eliminated from the proposed model. Detailed kinetic information about the thiourea–iodine reaction cannot be found in the literature, though Rábai and Beck have already shown that this reaction is extremely fast and cannot even be followed by a stopped-flow technique.^29^ They estimated that the rate coefficient of eq 5 is >2 × 10^4^ M^–1^ s^–1^. Our experiments indeed supported their recommendation (see Figure S2); therefore, we have fixed this value at 10^5^ M^–1^ s^–1^ throughout the whole calculation process. It should, however, be clarified that any value higher than this would have led to the same final results in the fitting procedure. Even though the first stage of the reaction was found to be quite rapid, with an excess of iodine one may easily observe a slower decay of iodine that can conveniently be measured by standard UV–vis spectroscopy. Evidently, the decrease in the iodine concentration may readily be attributed to the formamidine disulfide–iodine reaction, though it may not be the only possibility. It is also well-known that formamidine disulfide even under a slightly acidic condition is not so stable^56,57^ and ready to decompose into various products to be able to react further with iodine. Because detailed kinetic investigation of this system has not yet been performed according to the literature, we have studied in separate experiments the second phase of the thiourea–iodine reaction.

### Formamidine Disulfide–Iodine Reaction and Hydrolysis of Formamidine Disulfide

Figures 3–6 describe the effect of the reactants, pH, and initially added iodide ion on the absorbance–time profiles at the slower phase of the thiourea–iodine reaction. Not surprisingly, as found in the TDO–iodine system,^35^ the hydrogen ion as well as the iodide ion inhibits the reaction between formamidine disulfide and iodine.

On the basis of these observations, our calculations provided the following kinetic model (shown in Table 1) to describe the most important feature of the system. First, it should be emphasized again that according to the discussion mentioned in the previous subsection, not only the intermediate FDS but also the product of its hydrolysis (thiourea monoxide) is responsible for the relatively slow decay of iodine; thus, their kinetic models (hydrolysis of FDS and the FDS–iodine reaction) are inseparable in this study. This ascertainment is well supported by the experiments in which identification of the retention time of FDS clearly revealed that under our experimental conditions not only this species but also Tu and thiourea monoxide (TMO) appear in appreciable amounts, when FDS is dissolved in slightly acidic solutions (see Figure S1). It is therefore evident that the hydrolysis of FDS leading to the formation of thiourea and thiourea monoxide should be an indispensable part of the kinetic model (see step R19 in Table 1). Second, it should be mentioned that the acid dissociation constants of protonated FDS were found to be 5.49 (p*K*_1_) and 7.66 (p*K*_2_);^56^ thus, under our experimental condition, FDS almost exclusively exists in its doubly protonated form (FDSH_2_^2+^), which is why this species is involved in the given equilibrium. Our calculation has revealed that both the forward and reverse rate coefficients of this hydrolytic equilibrium can be determined precisely from the experiments as shown in Table 1. Upon comparison of these rate coefficients with the previously reported ones, it is easy to see that *k*_R19_ is in sound agreement with that (3.3 × 10^–4^ s^–1^) reported by Hu et al.^57^ The slight difference can easily be reconciled by the fact that the latter value was determined at a limited pH range (just at pH 2). However, there is a considerable difference between the *k*_–R19_ value of (9.32 ± 0.71) × 10^6^ M^–1^ s^–1^ presented here and that published previously (4.8 s^–1^).^57^ Although in the cited reference there is no direct information about the exact rate law of the reverse process, it is difficult to believe that it follows first-order kinetics with respect to just one of the reactants. It is much more conceivable that it is first-order with respect to both reactants; thus, the given value for that rate coefficient seems to be inappropriate in the given reference. Because the value for *k*_–R19_ given in Table 1 worked consistently well to describe the experiments reported here and could also be determined with a sound precision, we would rather keep it as determined here.

Step R20 is one of the feasible reactions for removing iodine in the presence of formamidine disulfide as a result of the hydrolytic product thiourea monoxide (TMO), where iodine oxidizes TMO via the formation of TDO and the iodide ion. It is well-known that thiourea trioxide does not react directly with iodine,^36^ while thiourea dioxide can react with iodine slowly via a sequence of reactions involving a halonium ion transfer process as an initiation.^35^ As is expected, the next oxide of thiourea in this row (thiourea monoxide) can be oxidized faster with iodine represented in step R20 of Table 1. Evidently, this reaction is a complex process, but our kinetic data do not provide further details to divide it into elementary reactions. Because preparation of pure thiourea monoxide is still an unresolved problem at present, we do not see any opportunity to study separately the thiourea monoxide–iodine reaction.

The other important reaction pathway for removing iodine in the presence of formamidine disulfide can be seen as step R21 in Table 1. Possibly, one of most surprising results of our calculations is that triiodide was found to be the reactive species with respect to formamidine disulfide to produce TMO and the iodide ion. To the best of our knowledge, this is the only sulfur-containing compound known so far for which iodine is less reactive than the triiodide ion in the given systems. This fact, however, can straightforwardly be reconciled by the appearance of the Coulomb attraction between oppositely charged reactants, which can easily enhance the reactivity of the triiodide ion over the neutral iodine species.

The third pathway responsible for removing iodine in the formamidine disulfide–iodine reaction is step R22. This is not a direct reaction, and actually thiourea formed from the hydrolysis of formamidine disulfide reacts with hypoiodous acid, which is always present in aqueous iodine solutions as a result of the well-known iodine hydrolysis. The rate coefficient of this reaction (*k*_R22_) was found to be (1.41 ± 0.12) × 10^9^ s^–1^, meaning that it could be determined quite precisely from our measured data.

Step R23 is also an important process and responsible for the appearance of elementary sulfur precipitation in the case of the hydrolysis of formamidine disulfide.^56^ As one can see in the absence of iodine, this pathway may become dominant, but under our experimental conditions, its effect is much less pronounced. To support this fact, elimination of this step would lead to only an increase from 1.51% to 1.70% in the average deviation, which may still be treated as acceptable. Even though the kinetic role of this step in this model is marginal, we would rather keep it in our model to be consistent with an earlier but independent report. At the same time, this reaction also provides further support for why the initial concentrations of the reactants were kept low; otherwise, the precipitation of sulfur would have easily disturbed the absorbance signals.

As Figures 3–6 show, the proposed kinetic model is working properly, though at least a paragraph is in order here to interpret unambiguously the inhibitory effect of hydrogen and iodide ions. In particular, the one involved iodide ion is difficult to see at first glance, when step R21 (reaction of FDS with the triiodide ion) in itself would suggest quite the opposite behavior. It is indeed evident that in itself step R22 should rather provide an autocatalytic effect of the iodide ion than an inhibitory one. However, step R22 is also crucial for removing iodine indirectly via hypoiodous acid. The amount of hypoiodous acid via step R4 decreases with increasing hydrogen and iodide ion concentrations. The combined kinetic effect of these two steps results eventually in an overall inhibition, meaning that the inhibitory effect of step R22 is more pronounced than the iodide catalysis of step R21 as expected by the comparison of *k*_R21_ and *k*_R22_ values.

### Thiourea–Iodate Reaction

The final step leading to the correct description of the kinetic curves measured in the thiourea–iodate system is to include the possible oxygen transfer reaction between the reactants to produce thiourea monoxide and iodous acid (see step R24). The best fit (average deviation found to be 1.51%) was achieved when the rate law of this step contained a hydrogen ion-independent and a H^+^-dependent term. Of course, additional fitting procedures were also performed when either of these terms was removed from the kinetic model. These calculations revealed that when *k*_R24_ and *k*_R24_^′^ were omitted average deviations of 1.88% and 1.70%, respectively, were obtained; meanwhile, the rest of the other parameters did not change significantly. Even though these models worked consistently well, we would keep both terms in the rate law just because not only the iodate ion but also iodic acid exists in the given pH range paving the way for proposing that both species be reactive toward thiourea. It should also be noted that the value of *k*_R24_ and *k*_R24_^′^ is consistent with that estimated by Rábai and Beck^29^ but significantly differs from that proposed by Wang et al.^30^

Last but not least, we should also mention that without step R25, the average deviation between the measured and calculated curves has never decreased below 6.0%, indicating that this reaction is also a crucial process. Many other reactions of the intermediates (HIO_2_, HOI, TMO, and FDS) have been considered without any improvement in the quality of the fit except for step R25, when the reaction of TMO with iodous acid was considered to produce TDO and HOI by a possible oxygen transfer process. We have found that the rate law of this reaction has to be proportional with [H^+^]; otherwise, the average deviation would increase significantly over 2.0%.

Figures 7–10 depict the effect of the reactants, hydrogen ion, and iodide ion on the measured and calculated kinetic curves. As one can clearly see, all of the most important trends are soundly described by the proposed kinetic model.

## Conclusion

In this work, the complex thiourea–iodate system was investigated and shown to have the peculiar kinetic feature of behaving as a dual-clock reaction. We have presented experimental evidence that the first kinetic stage of the reaction, formation of iodine, is delayed as long as substrate thiourea is present in the solution. Once it is completely consumed, iodine starts to build up rapidly to provide the unique characteristics of a substrate-depletive clock reaction.^22^ After this fast formation, the buildup of iodine stops for a while and the system apparently remains at this stage for a while as a result of the fine balance of the hydrolysis of formamidine disulfide, the FDS–iodine reaction, and the Dushman reaction. Once the concentration of FDS decreases to a certain level, one of the most important sinks of iodine ceases; thus, iodine starts to form more rapidly in a sigmoidal fashion as a result of the increasing importance of the TDO–iodate reaction having the characteristic autocatalytically driven clock behavior.^39^ It, therefore, serves as the main reason why the title system deserves to be designated as a dual-clock reaction.

Finally, it should also be emphasized that this study describes a general method for how the kinetics and mechanism of a kinetically complex reaction should be elucidated to remain coherent with kinetic models of its subsystems. At the same time, it provides additional support for why simultaneous evaluation of kinetic curves measured in the overall system along with its subsystem may be treated as a powerful method for obtaining comprehensive but feasible kinetic models.

## Supporting Information Available

Identification of HPLC peaks, results of stopped-flow experiments in the case of the first kinetic phase of the thiourea–iodine reaction, effect of aging on the title reaction, chemical structures of sulfur-containing species, and a figure depicting dual-clock behavior (PDF)
