# Supplementary material for: Compatible Kinetic Model for Quantitative Description of Dual-Clock Behavior of the Complex Thiourea–Iodate Reaction
Source: Inorg Chem. 2023 Jan 11;62(3):1192–201. doi: 10.1021/acs.inorgchem.2c03594 (PMC9875309; doi:10.1021/acs.inorgchem.2c03594)
Supplement: Supplementary file 1 — ic2c03594_si_001.pdf [file ic2c03594_si_001.pdf]

Supporting Information for Compatible Kinetic Model for Quantitative Description of Dual-Clock Behavior of the Complex Thiourea–Iodate Reaction

György Csekő,[†,¶] Qingyu Gao,[*,†] and Attila K. Horváth[*,‡]

†*School of Chemical Engineering, China University of Mining and Technology, Xuzhou 221116, People's Republic of China*

‡*Department of General and Inorganic Chemistry, University of Pécs, Ifjúság útja 6., H-7624 Pécs, Hungary*

¶*Current address: Department of General and Inorganic Chemistry, University of Pécs, Ifjúság útja 6., H-7624 Pécs, Hungary*

E-mail: gaoqy@cumt.edu.cn; horvatha@gamma.ttk.pte.hu

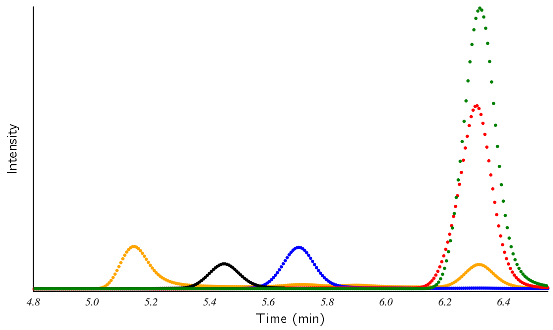

Figure S1: Identification of peaks appeared in the HPLC chromatograms. Iodate at 5.45 min (black), thiourea dioxide at 5.70 min (blue), thiourea at 6.29 min (green), iodide ion at 6.28 min (red), formamidine disulfide at 5.14 min (yellow). Please note that due to the unavoidable hydrolysis of FDS, the peak of thiourea as well as that of thiourea monoxide at 5.94 min (hardly visible) also appear on the chromatogram.

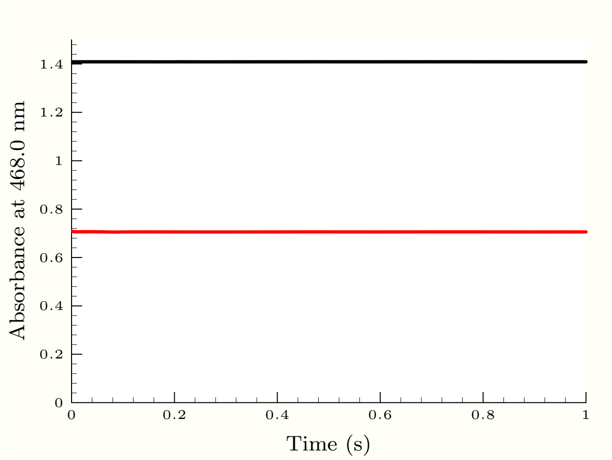

Figure S2: Result of the stopped-flow experiments when thiourea solution was mixed in a 1:1 ratio with a buffer solution in the absence (black) and presence (red) of iodine and iodide ion. Initial conditions are set as follows: $[\mathrm{Tu}]_0 = 1.8$ mM; $\mathrm{T}^0_{\mathrm{I}_2} = 1.81$ mM, $[\mathrm{I}^-]_0 = 50$ mM, pH = 1.55.

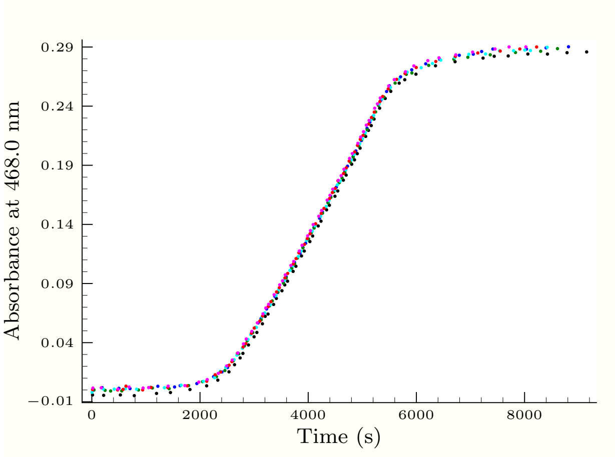

Figure S3: Effect of ageing on the kinetic curves in the thiourea–iodate reaction. Initial conditions are set as follows: $[\mathrm{Tu}]_0 = 0.45$ mM; $[\mathrm{IO_3}^-]_0 = 2.1$ mM, $[\mathrm{I}^-]_0 = 0$ mM, pH = 2.63. Aging time (s): 250 (black), 592 (blue), 802 (green), 998 (cyan), 1200 (red), 1405 (magenta).

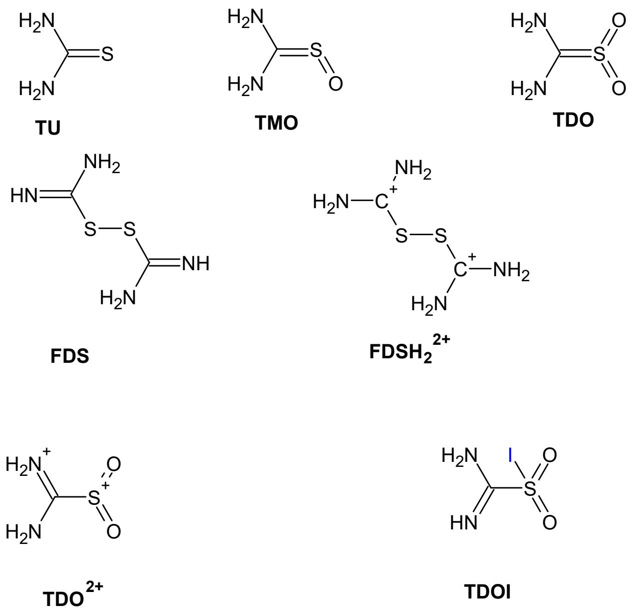

Figure S4: Chemical structure of key sulfur-containing species involved in the thiourea–iodate reaction.

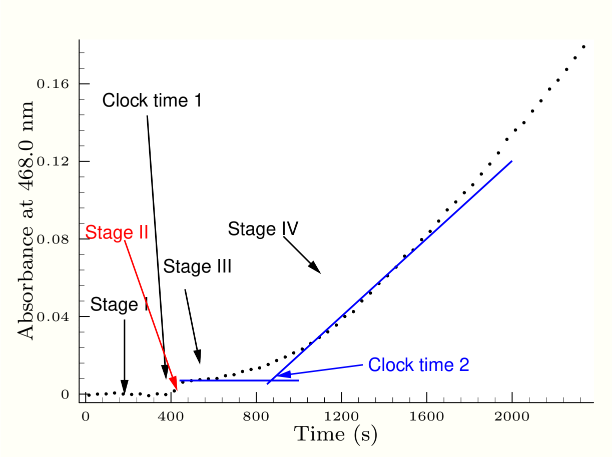

Figure S5: Measured absorbance–time curve in the thiourea–iodate reaction, where kinetic stages I, II and III and clock times are more visible. Experimental conditions are as follows: $[\mathrm{Tu}]_0 = 1.0$ mM, $[\mathrm{IO_3}^-]_0 = 2.8$ mM, pH = 2.63, $[\mathrm{I}^-]_0 = 0$ mM.
